# Overexpression of Tropomyosin Isoform Tpm3.1 Does Not Alter Synaptic Function in Hippocampal Neurons

**DOI:** 10.3390/ijms22179303

**Published:** 2021-08-27

**Authors:** Chanchanok Chaichim, Tamara Tomanic, Holly Stefen, Esmeralda Paric, Lucy Gamaroff, Alexandra K. Suchowerska, Peter W. Gunning, Yazi D. Ke, Thomas Fath, John Power

**Affiliations:** 1 Translational Neuroscience Facility, School of Medical Sciences, University of New South Wales, Sydney, NSW 2052, Australia; c.chaichim@unsw.edu.au; 2 Dementia Research Centre, Department of Biomedical Sciences, Faculty of Medicine, Health and Human Sciences, Macquarie University, Sydney, NSW 2109, Australia; tamara.tomanic@hdr.mq.edu.au (T.T.); holly.stefen@mq.edu.au (H.S.); esmeralda.paric@mq.edu.au (E.P.); lucy-sarah.gamaroff@students.mq.edu.au (L.G.); a.suchowerska@unsw.edu.au (A.K.S.); yazi.ke@mq.edu.au (Y.D.K.); 3 Cellular and Genetic Medicine Unit, School of Medical Sciences, University of New South Wales, Sydney, NSW 2052, Australia; p.gunning@unsw.edu.au

**Keywords:** actin cytoskeleton, tropomyosin, synapse function

## Abstract

Tropomyosin (Tpm) has been regarded as the master regulator of actin dynamics. Tpms regulate the binding of the various proteins involved in restructuring actin. The actin cytoskeleton is the predominant cytoskeletal structure in dendritic spines. Its regulation is critical for spine formation and long-term activity-dependent changes in synaptic strength. The Tpm isoform Tpm3.1 is enriched in dendritic spines, but its role in regulating the synapse structure and function is not known. To determine the role of Tpm3.1, we studied the synapse structure and function of cultured hippocampal neurons from transgenic mice overexpressing Tpm3.1. We recorded hippocampal field excitatory postsynaptic potentials (fEPSPs) from brain slices to examine if Tpm3.1 overexpression alters long-term synaptic plasticity. Tpm3.1-overexpressing cultured neurons did not show a significantly altered dendritic spine morphology or synaptic activity. Similarly, we did not observe altered synaptic transmission or plasticity in brain slices. Furthermore, expression of Tpm3.1 at the postsynaptic compartment does not increase the local F-actin levels. The results suggest that although Tpm3.1 localises to dendritic spines in cultured hippocampal neurons, it does not have any apparent impact on dendritic spine morphology or function. This is contrary to the functional role of Tpm3.1 previously observed at the tip of growing neurites, where it increases the F-actin levels and impacts growth cone dynamics.

## 1. Introduction

Most synapses between excitatory neurons form on dendritic spines, which are protrusions from dendrites that consist of a large, actin-rich head separated from the dendrite shaft by a thin neck. Actin exists in multiple populations in dendritic spines, with different functions and rates of turnover [1]. Most actin present in spines is dynamic and can change its organisation very rapidly [2].

The process of learning involves structural changes to synapses. The development and plasticity of dendritic spines are reliant on the flexibility of the actin cytoskeleton. The most studied cellular model of synaptic plasticity is long-term potentiation (LTP). In LTP, high-frequency stimulation causes a signalling cascade that leads to insertion of AMPA receptors, enlargement of dendritic spines, an increase in synaptic strength [3], and polymerisation of actin [4].

Blocking both actin polymerisation and depolymerisation separately [5,6] has been found to impair LTP, demonstrating that spine remodelling requires both processes at different stages. As well as overall spine shape modifications, the trafficking and anchoring of receptors at the membrane also require actin remodelling [7]. Overall, the actin cytoskeleton plays a key role in the function of dendritic spines.

Restructuring of actin is regulated by an assortment of actin-associated proteins. The tropomyosin family proteins act as gatekeepers for the access of other actin-associated proteins to actin. In mammals, there are four tropomyosin genes that produce over 40 different isoforms through alternative splicing [8]. They are differentially expressed at a cellular level and can segregate into various subcellular localisations within one cell type [8]. Most actin filaments are decorated with tropomyosin [9]. The various isoforms of tropomyosin produce subpopulations of actin with different properties.

Tpm3.1 is the major product of the TPM3 gene in neurons and is the most studied isoform. In developing mouse primary cultured neurons, it is enriched in the tips of neurites and filopodia [10]. In neurons, products of the TPM3 gene have been observed to localise to dendritic spines [11,12]. The presence of Tpm3.1 on actin filaments recruits myosin IIB, a force-generating molecular motor that has been implicated in maintaining the dendritic spine head width, activity in hippocampal neurons [13], and short-term memory [14]. It also increases inactive ADF/cofilin [10]. The actin depolymerising factor (ADF)/cofilin family of proteins binds to filamentous actin (F-actin) and depolymerises it into G-actin [15]. It is required to be activated for actin reorganisation and deactivated during spine stabilisation for memory consolidation [16]. Therefore, Tpm3.1 has an actin-stabilising function.

Experiments studying the effect of the overexpression of Tpm3.1 on neuronal development have found that it promotes growth: it increases the pool of F-actin in the growth cone of hippocampal neurons [17], as well as increasing the growth cone size [10], axonal branching, and the number and length of dendrites [17]. Tpm3.1-overexpressing neurons have also been found to overcome inhibition with Nogo-66 [18].

Pharmacological inhibition of Tpm3.1/2 has been found to decrease the uniformity of actin rings in axons, reduce the accumulation of proteins at the axon initial segment, and reduce the action potential firing frequency [19]. Knockout of Tpm3.1 reduces branching and the total neurite length in developing neurons [20].

While Tpm3.1 is present in the postsynaptic density (PSD) [12], its function there has not been previously studied. Here, we examined the role of Tpm3.1 in synaptic function and morphology using a transgenic mouse model that expresses human Tpm3.1 (hTpm3.1).

## 2. Results

### 2.1. The Expression of Tpm3.1 Is Highly Increased in Tpm3.1 Tg Mice and Is Enriched in Dendritic Spines

**Figure 1:**
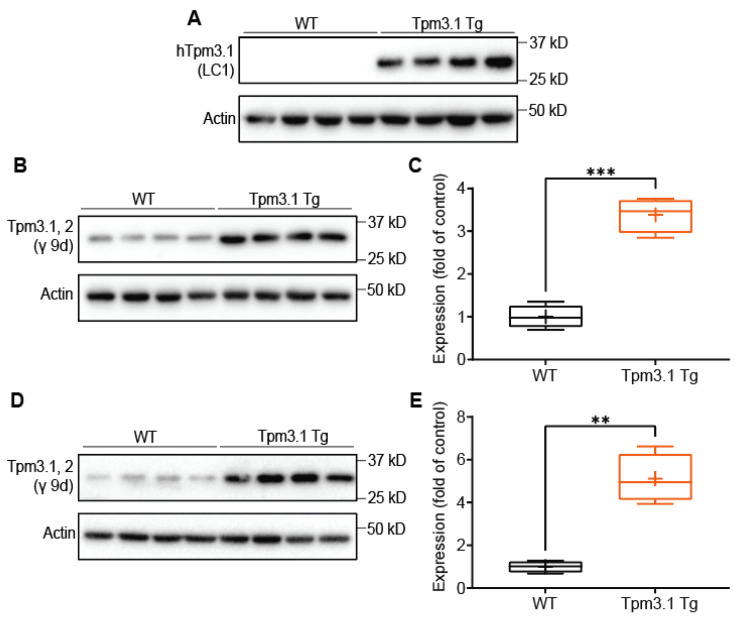
Expression of Tpm3.1 in brain homogenate and PSD of Tpm3.1 Tg mice, compared to WT mice. (**A**) Western blot using LC1 antibody shows expression of hTpm3.1 in transgenic mice and absence in WT mice. (**B**,**C**) γ 9d antibody for Tpm3.1 shows enrichment of Tpm3.1/2 in transgenic mice compared to WT in whole brain homogenate and (**D**,**E**) postsynaptic fraction. *n* = 4 mice per group. WT values are shown in black, Tpm3.1 Tg values shown in orange. ** indicates *p* < 0.01, *** indicates *p* < 0.001.

We first analysed the overexpression of Tpm3.1 in Tpm3.1-overexpressing transgenic mice (Tpm3.1 Tg). Brains were extracted from Tpm3.1 Tg and wild-type (WT) mice, prepared into a brain homogenate, and further processed to isolate the PSD. The expression level of Tpm3.1 was analysed using the LC1 antibody to detect exogenous human Tpm3.1 (hTpm3.1), and the γ 9d antibody to detect all Tpm3.1/2 [21]. As expected, WT mice did not express any hTpm3.1 (Figure 1A). Tpm3.1 Tg mice had an approximately threefold increase in Tpm3.1 in the total brain homogenate (Figure 1B,C), and a fivefold increase in the PSD-associated fraction (Figure 1D,E).

**Figure 2:**
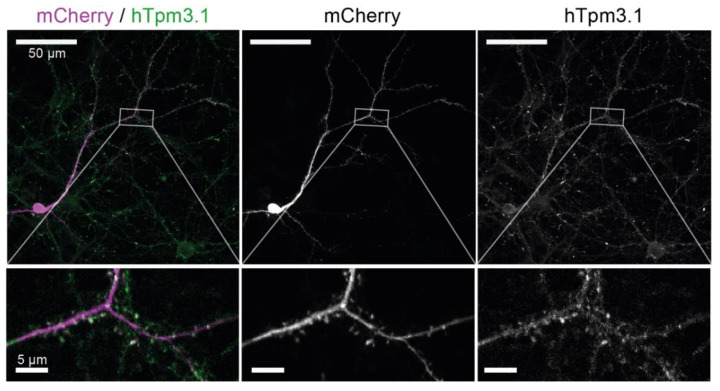
Staining for hTpm3.1 shows that it is enriched in dendritic spines. From left to right: merged image with mCherry in magenta and hTpm3.1 in green, mCherry channel, and hTpm3.1 channel.

To investigate the effect of Tpm3.1 overexpression, we used primary hippocampal neurons prepared from mouse embryos overexpressing Tpm3.1 and WT littermates. Cells were fixed and stained for hTpm3.1 to identify transgenic cells. We confirmed that, as with endogenous Tpm3.1 [12], hTpm3.1 is enriched in dendritic spines (Figure 2).

### 2.2. Tpm3.1 Overexpression Does Not Significantly Increase F-Actin in Dendritic Spines

**Figure 3:**
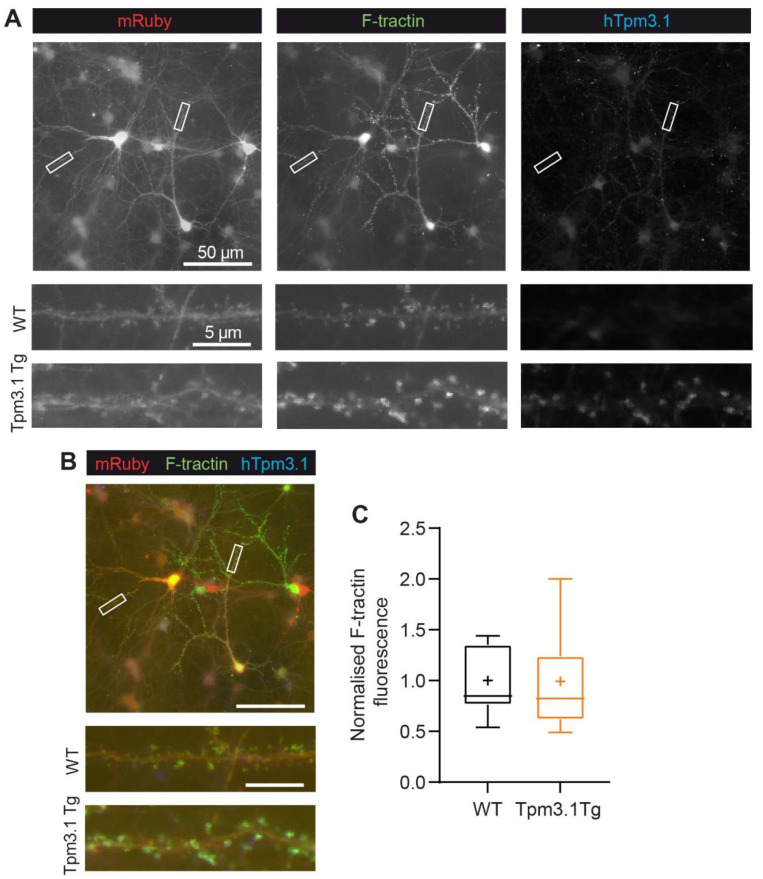
F-tractin measurement in dendritic spines. (**A**) Representative images of individual hTpm3.1, F-tractin, and mRuby channels and (**B**) merged image. (**C**) F-tractin fluorescence, WT *n* = 14, hTpm3.1 Tg *n* = 13 cells from 3 culture preparations. WT values are shown in black, Tpm3.1 Tg values shown in orange. Scale bars indicate 50 µm (top) and 5 µm.

To examine the effect of increased Tpm3.1 on actin in dendritic spines, we transduced neurons with cytosolic mRuby2 and F-tractin, a probe for F-actin fused with EGFP. Cells were fixed and immunostained for hTpm3.1 to identify transgenic cells, and spine F-tractin fluorescence was measured. The was little or no difference in spine F-tractin in transgenic and wild-type neurons (Figure 3C; WT: 1.0 ± 0.08; *n* = 14; Tpm3.1 Tg: 0.99 ± 0.14; *n* = 13, Mann–Whitney U = 79; *p* = 0.57).

### 2.3. The Effect of Tpm3.1 Overexpression on Dendritic Spine Morphology

We used a mixed culture system to study the effect of Tpm3.1 overexpression on spine morphology. Neurons were transfected to express fluorescent protein mCherry at 14 DIV, fixed at 17–18 DIV, and immunostained for hTpm3.1. Dendrite lengths of 20 µm were imaged, and individual protrusions from the dendrite were measured and counted.

**Figure 4:**
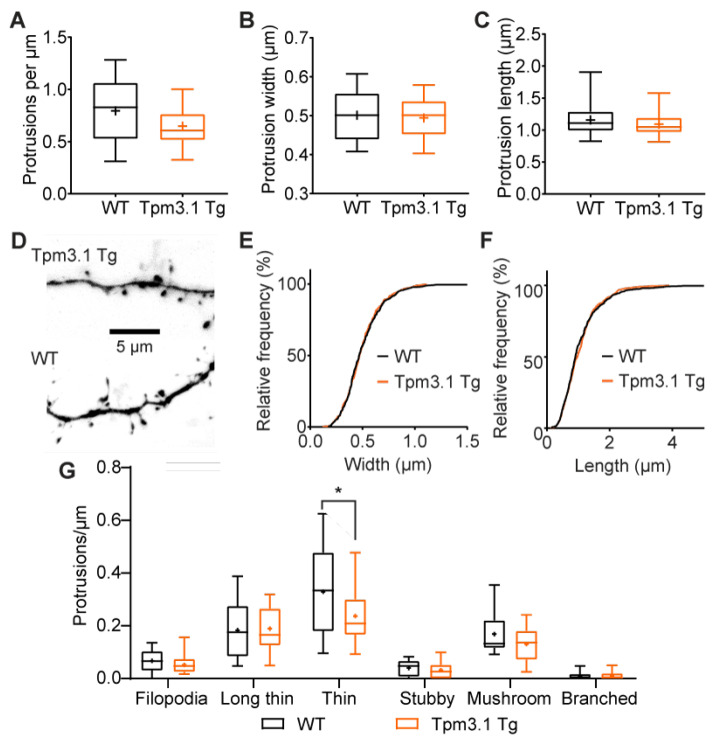
Overexpression of Tpm3.1 does not significantly alter dendritic spine morphology compared to WT. (**A**) Number of protrusions from the dendrite per μm was not significantly altered from control. (**B**) There was no observed difference in protrusion width or (**C**) average protrusion length. (**D**) Inverted representative images of dendritic segments, flattened from Z-stack. (**E**,**F**) Cumulative probability histograms of protrusion width and length showed no significant differences. (**G**) Categorisation of dendritic spine types in Tpm3.1 Tg cells. The length-to-width ratio of each dendritic spine was used to classify them as one of six types, shown here in increasing order of maturity. Tpm3.1 Tg cells had a lower density of thin spines, but no differences in any other category. A total of 456 spines from 14 Tpm3.1 Tg cells and 482 spines from 12 WT cells were analysed. WT values are shown in black, Tpm3.1 Tg values shown in orange. * indicates *p* < 0.05.

Overexpression of Tpm3.1 did not significantly change the dendritic spine density (Figure 4A; WT: 0.80 ± 0.09 µm^−1^, *n* = 12 cells; Tpm3.1 Tg: 0.65 ± 0.05 µm^−1^, *n* = 14; unpaired *t*-test *p* = 0.15). The protrusion width was not altered (Figure 4B; WT: 0.50 ± 0.02 µm, *n* = 12; Tpm3.1 Tg: 0.49 ± 0.02 µm, *n* = 14; unpaired *t*-test *p* = 0.77), nor was the protrusion length (Figure 4C; WT: 1.16 ± 0.08 µm, *n* = 12; Tpm3.1 Tg: 1.09 ± 0.05 µm, *n* = 14; Mann–Whitney test *p* = 0.46). Sorting spines into morphology categories based on their length-to-width ratio revealed that Tpm3.1 Tg cells had a lower density of thin spines (Figure 4G; Šídák’s multiple comparisons test; *p* = 0.02).

### 2.4. The Effect of Tpm3.1 Overexpression on Synaptic Function

To study basal synaptic function, we recorded mEPSCs from Tpm3.1-overexpressing cells. The frequency of events is taken to be a measure of the presynaptic release probability and synapse number, and the amplitude represents the strength of the synapses. Neurons had similar membrane resistances (WT: 162 ± 17 MΩ; Tpm3.1 Tg 157 ± 31 MΩ; *p* = 0.88), membrane capacitances (WT: 99 ± 5 pF; Tpm3.1 Tg: 90 ± 5 pF; *p* = 0.31), and access resistances (WT: 12 ± 1 MΩ; Tpm3.1 Tg 12 ± 1 MΩ; *p* = 0.94).

**Figure 5:**
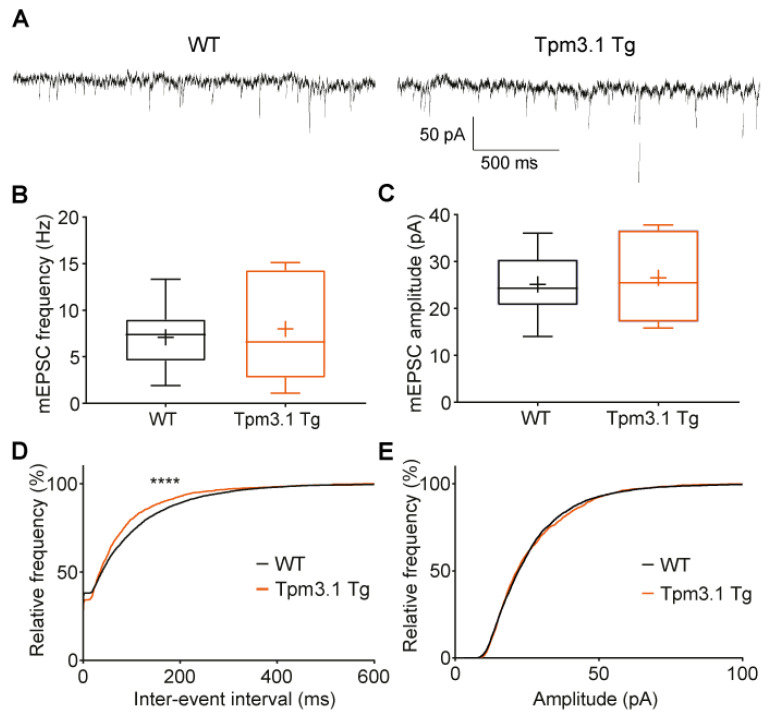
The effect of Tpm3.1 overexpression on mEPSC properties in cultured dissociated hippocampal neurons compared to WT. (**A**) Representative recording traces collected from WT and Tpm3.1 Tg cells. (**B**) Mean mEPSC frequency and (**C**) amplitude. (**D**,**E**) Cumulative probability histograms of mEPSC inter-event interval and amplitude; 200 events sampled from each cell. WT *n* = 21 cells, Tpm3.1 Tg *n* = 8 cells, from 4 separate culture preparations. WT values are shown in black, Tpm3.1 Tg values shown in orange. **** indicates *p* < 0.0001.

We did not observe a difference in the mean mEPSC frequency in Tpm3.1 Tg cells (Figure 5B; WT: 7.1 ± 0.64 Hz, *n* = 21; Tg: 7.99 ± 1.95 Hz, *n* = 8; *p* = 0.58) or the mean amplitude (Figure 5C; WT: 25 ± 1 pA; Tg: 26 ± 3 pA; *p* = 0.64). We plotted the cumulative probability distributions using the first 200 events from each cell and found that there was a significant shift in the inter-event interval (Figure 5D; Kolmogorov–Smirnov, *p* < 0.0001), showing that Tpm3.1 Tg cells were more likely to have shorter inter-event intervals, i.e., higher-frequency activity, but there was no difference in amplitude (Figure 5E; Kolmogorov–Smirnov, *p* = 0.20).

There were also no differences in the mEPSC rise time (WT: 0.82 ± 0.05 ms; Tg: 0.69 ± 0.04 ms; Mann–Whitney test *p* = 0.2) or decay (WT: 4.8 ± 0.470 ms; Tg: 4.0 ± 0.29 ms; Mann–Whitney test *p* = 0.32).

### 2.5. The Effect of Tpm3.1 on Long-Term Potentiation in Brain Slices

**Figure 6:**
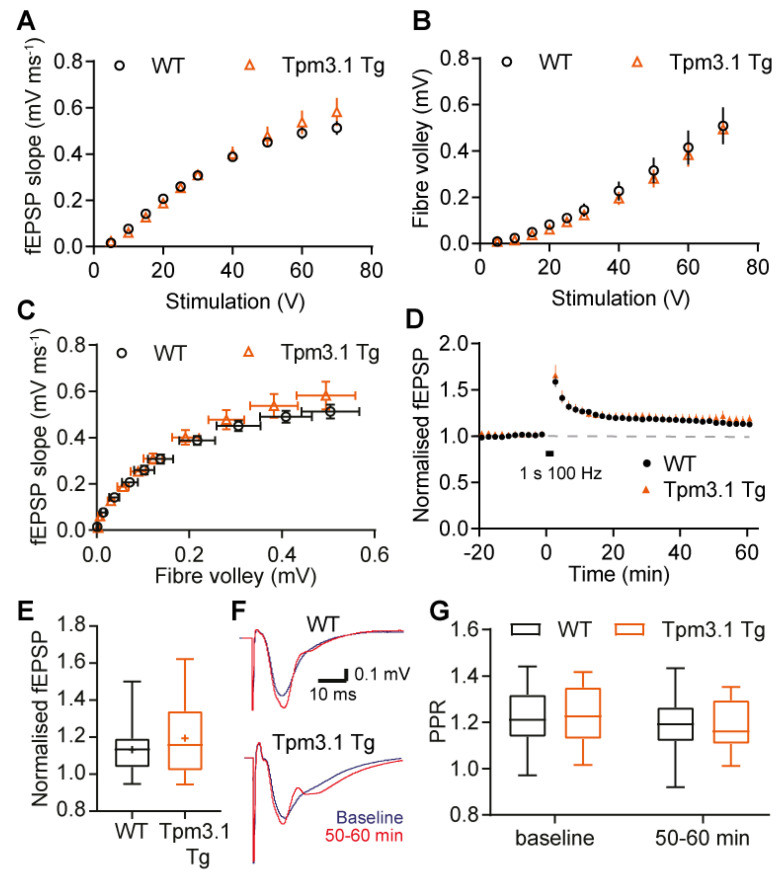
Tpm3.1 overexpression in acute hippocampal slices. (**A**) Input–output curve of maximum fEPSP slope plotted against stimulation voltage. (**B**) Input–output curve of fibre volley plotted against stimulation voltage. (**C**) fEPSP slope plotted against fibre volley to show synaptic efficacy. WT *n* = 30 slices from 20 mice, Tpm3.1 Tg *n* = 29 slices from 16 mice. (**D**) Plot of fEPSP over course of experiment, with each point representing the average from 2 min of recording. (**E**) Average normalised fEPSP from the last 10 min of recording after inducing LTP. (**F**) Example waveforms from baseline (blue), and last 10 min of recording (red). (**G**) PPR at baseline and last 10 min after HFS. WT *n* = 21 slices from 17 mice, Tpm3.1 Tg *n* = 19 slices from 14 mice. WT values are shown in black, Tpm3.1 Tg values shown in orange.

To examine the effect of Tpm3.1 overexpression on synaptic plasticity, we prepared acute brain slices from Tpm3.1 transgenic mice and recorded extracellular field potentials from the hippocampus (Figure 6). An input–output (IO) curve was generated, measuring the response size of the fEPSP slope and fibre volley at various levels of stimulation. From these IO curves, we measured the fEPSP slope, representing the synaptic current, and the fibre volley, representing the number of terminals activated by the stimulus. Plotted together, these show the synaptic efficacy, which is the postsynaptic response evoked per presynaptic stimulation.

We observed no difference in the paired pulse ratio (PPR) at baseline (Figure 6G; unpaired t-test; *t* = 0.31, *p* = 0.76), the fEPSP slope (Figure 6A; two-way RM ANOVA interaction *p* = 0.25, genotype *p* = 0.71), or the fibre volley (Figure 6B; two-way RM ANOVA interaction *p* ≥ 0.99, genotype *p* = 0.72). There was also no apparent difference in synaptic efficacy between the two groups (Figure 6C). Overall, basal synaptic function was not affected by Tpm3.1 overexpression in the brain slices.

The response was then recorded for 60 min (Figure 6D). We observed no difference between groups, both in the first 10 min and last 10 min of recording after LTP induction (Figure 6E; two-way ANOVA interaction *p* = 0.33, genotype *p* = 0.51). We also saw no difference in the PPR at any time point (Figure 6G; two-way RM ANOVA interaction *p* = 0.11, genotype *p* = 0.67). Together, these results suggest that overexpressing Tpm3.1 has no effect on synaptic plasticity.

## 3. Discussion

In this study, we examined the effect of Tpm3.1 overexpression on dendritic spine morphology and synapse function using transgenic mice that express hTpm3.1. Previous studies have shown that Tpm3.1 is enriched in dendritic spines [11,12]. Here, we show that hTpm3.1, expressed in transgenic cells, also localises to dendritic spines. Importantly, there was a fivefold increase in total Tpm3.1 in the PSD-associated fraction. These results suggest that overexpressed Tpm3.1 is properly trafficked.

In neurons, Tpm3.1 localises to several actin-rich compartments including the growth cone and axon initial segment [17,19]. Previous studies showed hTpm3.1 overexpression increases the size of actin-rich growth cones at the tip of growing neurites and the amount of F-actin present in growth cones [22]. While transgenic neurons had more Tpm3.1 associated with the postsynaptic specialisation, the amount of F-actin present in spines was not different from the control. There was also no apparent difference in the length and width of the spines. We observed a modest decrease in the number of thin spines in Tpm3.1 Tg cells. However, this decrease was not accompanied by a shift toward larger, more mature spine phenotypes, nor was there a difference in the spine density. Thus, unlike in growth cones, overexpression of Tpm3.1 in spines does not increase the F-actin pool or enlarge the spine. One possibility may be that the total actin filament level in dendritic spines is fixed and the excess Tpm3.1 remains in the soluble pool.

Despite its lack of effect on morphology, it is still possible for there to be an effect on synaptic function as actin dynamics are critical to receptor localisation [7]. In cultured neurons, Tpm3.1 overexpression did not alter the mean mEPSC amplitude or frequency. Spine volume is correlated with synaptic strength [23]. We noted a difference in the distribution of the inter-event intervals between Tpm3.1-overexpressing and wild-type cells; however, as there was no difference in the mean mEPSC frequency, this difference is difficult to interpret (Figure 5). Similar results were obtained in the brain slices. There was no apparent difference in the stimulus response function or the PPR measures of synaptic strength, and the transmitter release probability. This lack of difference in synaptic transmission is consistent with the lack of morphological differences.

Tpm3.1 is thought to have an actin-stabilising function [10], and thus overexpression may modulate the remodelling of actin in dendritic spines [24] associated with the induction of LTP. LTP is associated with a rapid increase in the spine head size to accommodate more receptors [23]. Our data suggest that Tpm3.1 does not play a key role in this process, in either the breakdown of the cytoskeleton or stabilising the new structure. There were no differences in the magnitude or time course of synaptic potentiation observed.

It is unclear why we did not detect any effects, given that studies of modulating Tpm3.1 expression in other compartments show significant changes. A possibility is that hTpm3.1 may not function normally in mouse cells, but this is unlikely given that past studies using the same model have observed effects. Furthermore, hTpm3.1 differs from mTpm3.1 by only one amino acid [21].

Previous studies have suggested there are functionally distinct populations of F-actin in dendritic spines [1,2]. These different populations are involved in maintaining the spine head size [1] and anchoring receptors to the membrane [7], and some are involved in trafficking receptors between membrane compartments [7,25]. It is possible that Tpm3.1 regulates postsynaptic F-actin populations which are involved in processes distinct from synaptogenesis and clustering of ionotropic glutamate receptors. It is also possible that the tropomyosins are not the rate-limiting step in these processes. It is known that actin filaments are already largely saturated with tropomyosin [9]. As we did not observe a change in the levels of F-actin in the postsynaptic compartment of Tpm3.1-overexpressing neurons, Tpm3.1 may also have functions beyond actin stabilisation in this compartment.

A detailed analysis of isolated PSD fractions from mouse brain tissue has revealed the presence of several tropomyosin isoforms [12], with the Tpm4 gene product Tpm4.2 being the most abundant Tpm isoform. Therefore, future studies will need to determine whether Tpm4.2 has a functionally distinct role compared to that of Tpm3.1 in regulating the spine shape and synaptic function of central nervous system neurons.

We demonstrated in our study that the Tpm3.1 transgenic model strongly overexpresses Tpm3.1 in the postsynaptic compartment. In other compartments (including the axon initial segment, growth cones, and dendritic tree), altering of Tpm3.1 expression leads to pronounced phenotypes. Contrary to this, our results surprisingly suggest the presence of a pool of filamentous actin, associated with the PSD, and defined by the decoration with Tpm3.1, which is not involved in synaptic function, as tested in our functional assays. Alternatively, the endogenous levels of Tpm3.1 in spines are already at saturation, the increased expression of exogenous Tpm3.1 remains in the soluble pool, and the association of Tpm3.1 with the PSD is independent of Tpm3.1′s association with actin. Future studies will be important in determining the subspine localisation of Tpm3.1 to provide further insight into which filament populations in dendritic spines are decorated with Tpm3.1.

## 4. Materials and Methods

### 4.1. Animals

All animal studies were carried out in accordance with the New South Wales Animal Research Act and Regulation and approved by the Animal Ethics Committees of UNSW Sydney and Macquarie University. Mice were housed in a temperature-controlled facility (22–24 °C) on a 12 h light–dark cycle. The original Tpm3.1 transgenic mouse line was generated on an FVB/NJ background as described in [10] and was previously backcrossed to a C57BL/6 background as described in [26]. The mice express hTpm3.1 under the β-actin promoter, resulting in ubiquitous expression of the transgene.

### 4.2. Genotyping

Tpm3.1-overexpressing mice were screened for the presence of the human Tpm3.1 gene. Here, 2 mm tail samples from each mouse were digested in 200 μL of an alkaline lysis buffer (25 mM NaOH and 0.2 disodium EDTA in water) at 95 °C for 1 h. An amount of 200 μL of neutralising buffer (40 mM Tris-HCl in water) was then added to each sample before centrifuging at 13,800 rpm for 15 min. Samples were stored at 4 °C until being required. DNA was amplified by a polymerase chain reaction. An amount of 0.5 μL of DNA sample was added to 10 μL 2× EconoTaq Plus Green (Lucigen, Middleton, WI, USA) and 0.25 mM of each primer in water for a total volume of 19.5 μL. Forward and reverse primer sequences were, respectively: AGCCAAGCTGGAAAAGACAA and ATGCTATCACCTCCCCTGTG. The mixture was heated to 94 °C for 3 min, and then it underwent 30 cycles of amplification consisting of 30 s at 94 °C, 60 s at 60 °C, and 45 s at 72 °C in a thermocycler (Mastercycler epgradient S, Eppendorf, Hamburg, Germany). The amplified product was then separated by gel electrophoresis in 1% agarose gel and stained with SYBR Safe (Invitrogen, Carlsbad, CA, USA). The gel was visualized under ultraviolet light (Geliance 200 visualisation system, PerkinElmer, Waltham, MA, USA). The expected product size for transgenic animals was approximately 200 base pairs.

### 4.3. Quantification of Tpm3.1 Expression

Quantification of Tpm3.1 expression was performed as previously described [12]. Whole brains were removed from mice and snap frozen. The brain homogenate was prepared by homogenising with buffer (0.1 mM EDTA, 0.1 mM EGTA, 0.25 mM PMSF, and 1 mM HEPES at pH 7.4). The homogenate was centrifuged twice at 1000× *g* for 7 min, and some supernatant was collected for the total brain homogenate measurement. The rest was further processed to collect synaptosomes, which were isolated as described in [27]. The synaptosome fraction was then used to prepare the PSD fraction as described by [28].

Western blotting was performed as previously described [12]. Membranes were blocked in 5% milk powder or BSA in 0.1% Tween-TBS and probed with primary antibodies overnight at 4 °C. The primary antibodies used were the LC1 antibody for hTpm3.1 and the γ 9d sheep polyclonal antibody for all Tpm3.1s, which have been previously characterised in detail in [21]. Membranes were then washed with 0.1% Tween in TBS, and HRP-conjugated secondary antibodies (GE Healthcare, Sydney, Australia) were applied. Membranes were developed using Luminata Crescendo Western HRP Solution (Millipore) and imaged using the GelDoc system (BioRad). Quantification was performed by densitometry using ImageJ (version 1.47).

### 4.4. Cell Culture

Mouse dissociated hippocampal cultures were prepared as previously described [29]. Heterozygous transgenic mice were mated. On embryonic day 16.5, pregnant mice were sacrificed by cervical dislocation. Embryos were harvested and placed in a 6 cm dish with ice-cold 1x HBSS to induce hypothermia. A cut was made along the midline of the head, and the brain was removed using forceps. The brain was hemisected, and the meninges were peeled off. The hippocampus was isolated using microscissors. Isolated hippocampal tissue was placed in 2 mL 1× HBSS with 250 µL trypsin (Sigma-Aldrich, Sydney, Australia) at 37 °C for 20 min. An amount of 250 µL Deoxyribonuclease (DNaseI, Sigma, Sydney, Australia) was added for 30 s before being diluted with DMEM with 10% FBS. The total volume of the tissue solution was adjusted to 1 mL, and the tissue was very slowly triturated with fire-polished glass Pasteur pipettes until homogenous. The volume of the solution was adjusted up to 10 mL with DMEM with 10% FBS, and debris was left to settle in the bottom of the tube. The supernatant was centrifuged at 0.3 RCF for 7 min, and the pellet was resuspended in DMEM and 10% FBS. Cells were plated on 12 mm coverslips (#1.5 thickness glass, Menzel) coated with 100 µg/mL poly-D-Lysine (Sigma-Aldrich) at a density of 14 × 10^4^ cells/mL. Media were changed to 1 mL per well of Neurobasal (Life Technologies) with 2% B27 supplement and 0.25% Glutamax (Invitrogen) 2 h after plating. Cells were maintained at 37 °C with 95% oxygen and 5% CO_2_.

### 4.5. Plasmids and Cloning

All AAV constructs were cloned using NEBuilder Hifi DNA Assembly Master Mix (New England Biolabs, E2621L). AAV pCAG-mRuby2 was obtained from Addgene (plasmid #89686, a kind gift from Wilson Wong). The F-tractin sequence was amplified from pEGFP-C1 F-tractin-EGFP Addgene plasmid #58472 using the following primers: forward 5′-gctcgcgactagtcgattcgcaccatggcgcgaccacggggc-3′ and reverse 5′-cctt-gctcactgatcctaatcctgaccctgcggccgctgcggc-3′. The EGFP sequence was amplified out of pEGFP-N1-ACTR3 Addgene plasmid #8462 using the following primers: forward 5′-tcaggattaggatcagtgagcaagggcgaggag-3′ and reverse 5′-tatcgataagcttgatatcgttactt-gtacagctcgtccatg-3′. These primers put the Kozak sequence in front of the F-tractin sequence, as well as placing the linker Ser-Gly-Leu-Gly-Ser in between the two amplicons. Both F-tractin and EGFP amplified fragments were cloned into an AAV target vector at the EcoRI restriction site. The AAV target vector used contains 1.1 kb chicken β-actin promoter (CBA), bovine growth hormone polyadenylation element (bGHpA), and woodchuck hepatitis virus posttranscriptional regulatory element (WPRE), flanked by AAV2 inverted terminal repeats (ITRs), and was previously described in [30].

### 4.6. Viral Production and Viral Transduction of Cultured Neurons

AAV_CAG-mRuby2, AAV_F-tractin-EGFP, and AAV_hTpm3.1-IRES-mRuby2 were packaged into the PHP.B capsid, and adeno-associated virus production was performed as previously described [30]. Briefly, 293T cells were seeded at 70–80% confluence in complete DMEM (Sigma, Sydney, Australia) with 10% FBS. At 3 h before transfection, culture medium was replaced with IMDM (Sigma, Sydney, Australia). Transfection was performed with AAV genome-containing plasmid, pFdelta6, and AAV-PHP.B using polyethyleneimine-Max (PEI-Max, Polysciences) reagent. Cell harvesting was carried out 72 h after transfection, while clarification of the supernatant was conducted with 40% PEG8000/2.5 M NaCl to a final concentration of 8% PEG8000/0.5M NaCl and incubated at 4 °C for at least 2 h, after which it was centrifuged at 2000× *g* for 30 min. For treatment of the combined precipitate from the clarified supernatant and cell pellet, sodium deoxycholate (0.5% final concentration) and benzonase (500 U) were used at 37 °C for 40 min. Purification of supernatants was performed using ultracentrifugation in an iodixanol gradient (475,900× *g* for 2 h at 18 °C). Concentrating and exchanging of AAV particles were carried out in PBS in an Amicon 100 kDa 15 mL concentrator at 5000× *g* at 4 °C. Genomic titering was conducted by quantitative polymerase chain reaction (qPCR), and the following titres were obtained: AAV-PHP.B_mRuby2 (2.2 × 10^14^ vp/µL) and AAV-PHP.B_F-tractin-EGFP (1.44 × 10^14^ vp/µL). The viral stock solution was diluted in Neurobasal media, and 8 × 10^8^ viral particles per 70,000 hippocampal cells were pipetted directly into the cell culture wells at 3–5 DIV.

### 4.7. mEPSC Recordings

Cells were recorded at 17–18 DIV. Coverslips were perfused with extracellular solution (110 mM NaCl, 10 mM HEPES, 10 mM glucose, 2 mM CaCl_2_, 0.8 mM MgCl_2_, 5 mM KCl) at room temperature using a peristaltic pump (Minipuls 3, Gilson, Middleton, WI, USA). After a cell was patched, the perfusion was switched to a separate 20 mL aliquot of extracellular solution with 0.5 µM TTX and 100 µM picrotoxin. Patch electrodes were made from thin-walled borosilicate capillaries, 1.2 mm OD, 0.94 ID, 100 length (Harvard Apparatus), and pulled using a Narishige Model PC-10 microelectrode puller to a tip resistance of 3–5 MΩ. Electrodes were filled with internal solution (110 mM caesium methanesulfonate, 8 mM NaCl, 10 mM HEPES, 2 mM Mg_2_ATP, 0.3 mM Na_3_GTP, 0.1 mM spermine tetrahydrochloride, 7 mM phosphocreatine, 10 mM EGTA, 5 mM CsCl) with 50 µM Alexa Fluor 594 and filtered through a 0.22 µm syringe-driven filter unit (Millex). Recordings of mEPSCs were performed at a holding potential of −70 mV with an Axopatch 200B amplifier, filtered at 2 kHz, digitised at 5 kHz with a Digidata 1440A, and saved with Clampex 10.2 (Molecular Devices, San Jose, CA, USA).

After five minutes, stable recordings were obtained from a cell, and the patch electrode was left on for as long as possible to allow the dye to enter the cell. An image was taken of the cell for identification after immunocytochemistry for hTpm3.1. After 1–2 h of recording, the coverslip was fixed in 4% PFA for 15 min at room temperature and then washed three times with 1× PBS.

mEPSCs were detected and measured using AxoGraph (Sydney, Australia). A notch filter (49.9–50.1 Hz) was applied, and an event template (maximum 0.5 ms rise time, 3 ms minimum decay time) was used to collect mEPSCs [31]. Events outside of 5–150 pA were excluded, and detected events were manually verified. Event amplitudes and inter-event intervals were measured. Either 5 min of activity or 1000 events were analysed from each cell. Out of 72 cells that were recorded, 40 were recovered. A further 11 cells were excluded because the recordings were too unstable to be able to identify mEPSCs.

### 4.8. Transfection

Tpm3.1 Tg mixed cultures were transfected with pmCherry-C1 (Clontech #632524). Transfection of primary cultures was performed at 14 DIV using Lipofectamine 3000 reagent. Amounts of 1.32 μg plasmid DNA and 2.56 μL Lipofectamine 3000 were used per coverslip. The required amount of Lipofectamine was mixed with half of the total required Neurobasal media in one tube. Plasmid DNA and the other half of the media were combined in a second tube. The Lipofectamine/medium was then pipetted dropwise into the DNA tube and mixed gently by inverting. It was left to incubate at room temperature in the biosafety cabinet for 25 min. All media were collected from the wells and transferred to a 15 mL tube. An amount of 50 μL of the original medium was added back to each well, along with 100 μL of transfection mixture. The cells were placed in an incubator for 60 min, along with cultured media. After the incubation period, the transfection media were removed, and the collected cultured media were returned to each well. The cells were fixed for 15 min in 4% PFA, 4 days later at 18 DIV, to be immunostained for identification of cells from Tpm3.1 transgenic embryos.

### 4.9. Immunocytochemistry

Unstained coverslips were stored wrapped in Parafilm at 4 °C for no more than 2 weeks before being processed. Cells were permeabilised with 0.1% Triton X100 in PBS for 5 min and blocked with 2% FBS in PBS for 30 min. They were incubated with the primary antibodies mouse LC1 [21] (at 1:200 dilution in blocking solution) and rabbit GFP antibody (1:500 dilution in blocking solution; Abcam ab290), for 2 h at room temperature or overnight at 4 °C. Cells were then washed 5 times with 1× PBS and then incubated with secondary antibodies goat anti-mouse Alexa 488 (1:500 dilution in blocking solution, Life Technologies, A32723) and goat anti-rabbit Alexa 647 (1:250 dilution in blocking solution, Life Technologies, A32733) for 30 min at room temperature, protected from light. They were then washed again with 1× PBS 5 times and dipped in Milli-Q element purified water before being mounted on glass slides using Fluoromount (Sigma-Aldrich, Sydney, Australia) mounting medium. The slides were left to set in the dark overnight. Clear nail polish was then used to seal the edges of the coverslips.

### 4.10. Identifying Transgenic Cells

Coverslips were viewed on an LSM 710 confocal microscope (Zeiss) using a 20 × 1.0 NA water immersion objective or a 63 × 1.4 NA oil immersion objective and an Axio-scope (Zeiss). Alexa 488 was excited with a 488 nm argon ion laser, and emission was captured at 493–581 nm. For each batch of immunostained coverslips, we imaged an area that clearly contained both hTpm3.1-positive and negative cells so they could be distinguished. To preserve consistency, imaging conditions and exposure times were kept the same across all biological replicates. Patched cells were identified by referring to images taken at the time of patching.

### 4.11. Dendritic Spine Morphology Analysis

Immunofluorescence images were taken using an LSM 710 confocal microscope (Zeiss) with a 63 × 1.4 NA oil immersion objective. mCherry was excited with a 561 nm DPSS laser, and emission was captured at 578–696 nm. Two 20 μm length segments of secondary dendrites 50–75 μm away from the soma were selected from each neuron. A Z-stack image with a 0.21 μm interval and 0.04 × 0.04 μm pixel size was acquired from each selected dendrite.

Deconvolution was performed on images using the DeconvolutionLab2 plugin in ImageJ [32]. Images were processed with 5 iterations of the Richardson–Lucy algorithm using a point spread function generated by the Diffraction PSF 3D plugin (Optinav). Spine morphology was analysed as previously described [33]. Z-stack images were imported to RECONSTRUCT [34]. The length of the dendrites and the length and widths of spines were manually traced and measured. Spine density, average protrusion width, and average protrusion length for each cell were calculated.

### 4.12. Dendritic Spine Actin Quantification

Images were taken using an Axio Imager (Zeiss) with a Plan-Apochromat 63×, 1.4 NA, 0.19mm WD Oil objective. EGFP, mRuby2, and Alexa 647 were excited with a Xenon HXP lamp at 488 nm, 555 nm, and 647 nm, respectively, and captured using filters FITC (Semrock #3540B-zero) BP465-500/BS506/BP516-556, Cy3 (Semrock #4040B-zero) BP511-551/BS562/BP573-613, and Cy5 (FS#50) BP625-655/BS660/BP665-715. Exposure times were kept consistent across all biological replicates for consistency purposes at 130 ms for EGFP, 460 ms for mRuby2, and 28 ms for hTpm3.1.

F-actin fluorescence was quantified in ImageJ (v. 2.1.0). Cells transduced with both AAV_mRuby2 and AAV_F-tractin-EGFP were selected. F-tractin has been previously confirmed as a reliable reporter of F-actin levels in cultures of primary neurons without a significant effect of the reporter on cell function [35]. Tpm3.1 Tg cells were distinguished from WT cells based on the LC1-Alexa 647 fluorescence level. Polygon selections were conducted to outline dendritic spines, and the mean fluorescence intensity in the F-tractin-EGFP channel was measured. A nearby area of the background was measured and subtracted from the spine fluorescence. To control for differences in the fluorescence level between experimental sets, F-actin fluorescence, for each set, was normalised to the mean wild-type fluorescence of that set. More than 25 spines were sampled from at least 2 segments of the dendritic tree for each neuron.

### 4.13. Brain Slice Preparation

Brain slices were prepared from 6–8-week-old male and female mice according to standard procedures. Mice were anaesthetised using isoflurane and decapitated, and the brain was removed and placed in ice-cold modified artificial cerebrospinal fluid (ACSF), containing 124 mM sucrose, 62.6 mM NaCl, 2.5 mM KCl, 26 mM NaHCO_3_, 1.2 mM NaH_2_PO_4_, 10 mM glucose, 0.5 mM CaCl_2_, and 3.3 mM MgCl_2_. The ventral surface was then affixed to the cutting platform using a cyanoacrylate adhesive.

Horizontal slices (400 μm) were cut using a vibratome (model VT1200, Leica, Wetzlar, Germany). During cutting, slices were transferred from the vibratome chamber to a holding chamber containing standard ACSF (124 mM NaCl, 3 mM KCl, 26 mM NaHCO_3_, 1.2 mM NaH_2_PO_4_, 10 mM glucose, 2.5 mM CaCl_2_, 1.3 mM MgCl_2_) at room temperature. The solution was continuously infused with 95% O_2_/5% CO_2_. Slices were left to recover for at least 1 h before recording. Slices were used within 7 h of cutting, corresponding to the optimal period of slice health [36].

### 4.14. fEPSP Recordings

Slices were transferred individually to the tissue recording system (Kerr Scientific Instruments, Christchurch, New Zealand) and continuously perfused with standard ACSF at room temperature. A bipolar, Teflon-coated tungsten stimulating electrode (Kerr Scientific Instruments) was placed in the stratum radiatum, aligned to the end of the dentate gyrus, and the recording electrode was placed approximately 800 μm from the stimulating electrode.

Stimuli were delivered via an isolated stimulator (model DS2, Digitimer, Hertfordshire, England, or A-M Systems, Model 2200, Washington, DC, USA), triggered through a Powerlab (model 4/2ST, AD Instruments, Sydney, Australia). Field potentials were amplified at 100× using a KSI Tissue Recording System Amplifier (Kerr Scientific Instruments, Christchurch, New Zealand) and digitised with the Powerlab. Traces were acquired using Scope (AD Instruments, Sydney, Australia).

A stimulus response curve was conducted by varying the stimulus intensity from 5 to 70 V, using the following step sequence: 5, 10, 15, 20, 25, 30, 40, 50, 60, and 70 V. Slices were discarded if the maximum fEPSP amplitude was below 0.6 mV. The stimulus intensity eliciting 50% of the maximum amplitude was identified, and field potentials were evoked in pairs with a 50 ms interval at this stimulus intensity at 30 s intervals. After obtaining a stable baseline for 20–30 min, LTP was induced with 1 burst of high-frequency stimulation (100 Hz 1 s). Responses were then recorded for the following 60 min.

### 4.15. fEPSP Analysis

Electrophysiological data were analysed offline using AxoGraph (Sydney, Australia). Measures included the fibre volley amplitude, the fEPSP slope, and the paired pulse ratio. Fibre volley amplitude was defined as the amplitude of the negative peak preceding the fEPSP. The fEPSP slope was defined as the maximum slope during the initial 2.5 ms after the fibre volley. This was used to measure excitatory activity. The paired pulse ratio was calculated as the second fEPSP slope divided by the first, in order to provide a measure of the presynaptic transmitter release probability [37].

### 4.16. Statistical Analysis

Statistical analysis was carried out using GraphPad Prism (GraphPad Software, La Jolla, CA, USA). Differences between groups were tested as indicated with Student’s t-test, the Mann–Whitney test, the Kolmogorov–Smirnov test, 2-way ANOVA with Šídák’s multiple comparisons test, or mixed model analysis with α = 0.05. Box–whisker plots indicate the median, quartiles, minimum, and maximum. Data are shown as mean ± standard error of the mean. Data collection and analysis were performed blind to the genotype of the animals or cells.

## Data Availability

All relevant data are provided within the manuscript.
